# Molecular basis for governing the morphology of type-I collagen fibrils by Osteomodulin

**DOI:** 10.1038/s42003-018-0038-2

**Published:** 2018-04-19

**Authors:** Takumi Tashima, Satoru Nagatoishi, Jose M. M. Caaveiro, Makoto Nakakido, Hiroshi Sagara, Osamu Kusano-Arai, Hiroko Iwanari, Hitomi Mimuro, Takao Hamakubo, Shin-ichi Ohnuma, Kouhei Tsumoto

**Affiliations:** 1 Department of Chemistry & Biotechnology, School of Engineering, The University of Tokyo, Tokyo, 108-8639 Japan; 2 Department of Bioengineering, School of Engineering, The University of Tokyo, Tokyo, 108-8639 Japan; 3 Project Division of Advanced Biopharmaceutical Science, The Institute of Medical Science, The University of Tokyo, Tokyo, 108-8639 Japan; 4 Medical Proteomics Laboratory, The Institute of Medical Science, The University of Tokyo, Tokyo, 108-8639 Japan; 5 Laboratory of Global Healthcare, Graduate School of Pharmaceutical Sciences, Kyushu University, Fukuoka, 812-8582 Japan; 6 Quantitative Biology and Medicine, Research Center for Advanced Science and Technology (RCAST), The University of Tokyo, Tokyo, 153-8904 Japan; 7 Department of Infection Microbiology, Research Institute for Microbial Diseases, Osaka University, Osaka, 565-0871 Japan; 8 Department of Infectious Diseases Control, International Research Center for Infectious Diseases, Institute of Medical Science, The University of Tokyo, Tokyo, 108-8639 Japan; 9 Institute of Ophthalmology, University College London (UCL), London, EC1V 9EL UK

## Abstract

Small leucine-rich repeat proteoglycan (SLRP) proteins have an important role in the organization of the extracellular matrix, especially in the formation of collagen fibrils. However, the mechanism governing the shape of collagen fibrils is poorly understood. Here, we report that the protein Osteomodulin (OMD) of the SLRP family is a monomeric protein in solution that interacts with type-I collagen. This interaction is dominated by weak electrostatic forces employing negatively charged residues of OMD, in particular Glu284 and Glu303, and controlled by entropic factors. The protein OMD establishes a fast-binding equilibrium with collagen, where OMD may engage not only with individual collagen molecules, but also with the growing fibrils. This weak electrostatic interaction is carefully balanced so it modulates the shape of the fibrils without compromising their viability.

## Abstract (web summary)

Takumi Tashima and colleagues provide structural insights into how collagen fibrils are shaped by Osteomodulin. Osteomodulin keeps a fast-binding equilibrium with the collagen fibrils to slow down its growth, promoting the formation of uniform, intact collagen fibrils.

## Introduction

The extracellular matrix (ECM) has important roles in organizing tissues and regulating cell behavior. Dysregulation of ECM structure causes several diseases, such as fibrosis, favoring cancer progression^1,2^. Type-I collagen is a major component of ECM and the most abundant protein in our body. Collagen assembles into fibrils after secretion from cells contributing to tissue strength and regulating cell behavior through various signal-transduction pathways^3^. The shape and/or the diameter of collagen fibrils are carefully regulated in each tissue having specific roles for optimal tissue strength^4,5^. Over the years, researches have unveiled a number of proteins that influence the size of collagen fibrils, such as collagen type V or small leucine-rich repeat proteoglycans (SLRPs)^1,3,6^. However, it is unclear how the regulatory mechanism works at the molecular level.

SLRPs comprise an important family of proteins, divided into five subclasses, that influence collagen fibril formation^6^. Class I SLRPs is exemplified by the protein Decorin (DCN) affecting collagen fibril formation^7^. A study employing a DCN knock-out mouse has shown that the skin of these mice exhibits an irregular structure by the disruption of the shape of the collagen fibrils^8^. Class II SLRPs such as Lumican and Fibromodulin are also known to be involved in the formation of collagen fibrils^9,10^. Recently, our group has demonstrated that Osteomodulin (OMD), a member of class II SLRPs, regulates the diameter and shape of collagen fibrils^11^. However, we are far from understanding how SLRPs, including OMD, regulate collagen fibrils at the molecular level. Although molecular dynamics simulations have been employed to emulate the binding mechanism between SLRPs and collagen^12^, there is no verification from an experimental point of view.

Here, we report that OMD exists as a monomer in solution. Biophysical analysis of the interaction between collagen molecules/fibrils and monomeric OMD was carried out to understand its regulatory mechanisms in collagen assembly. We revealed the binding site to collagen and its driving force, suggesting a mechanism that explains the role of OMD in fibril formation.

## Results

### Structural analysis of OMD

**Fig. 1:**
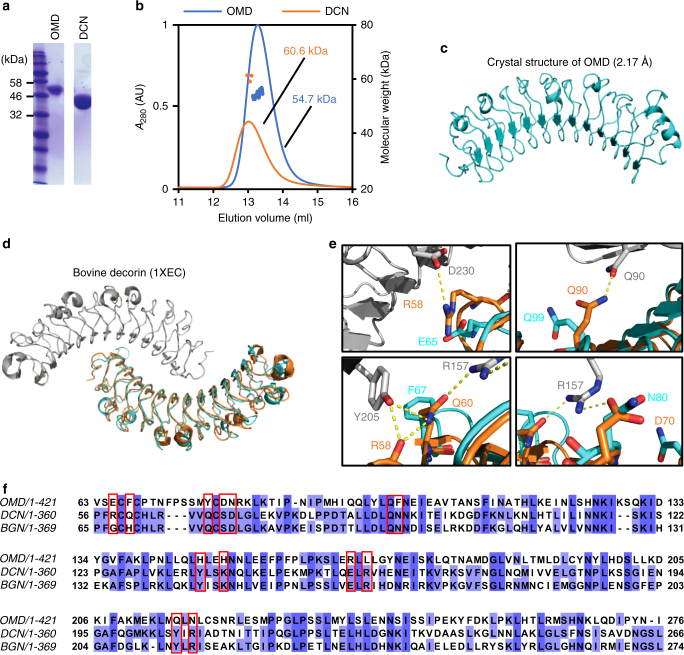
Physicochemical properties of OMD. **a** SDS-PAGE of OMD and DCN. **b** SEC-MALS of OMD and DCN. Solid lines represent absorbance at 280 nm (blue: OMD, orange: DCN). Dotted lines represent molecular weight (blue: OMD, orange: DCN). **c** X-ray crystal structure of OMD. The N-terminal and C-terminal regions of OMD are depicted on the left and right side of the panel, respectively. **d** Superimposition of OMD (cyan) on DCN dimer (gray and orange). The structure of DCN was obtained from the PDB (entry code 1XEC). **e** Interface residues at the DCN dimer (gray and orange). The structure of OMD (cyan) has been superimposed on one of the chains of the DCN dimer. The figure evidenced the different character of several residues of OMD at the position where residues of DCN make important contributions to the dimer interface. Yellow dotted lines represent H-bonds between molecules of DCN. **f** Sequence alignment between OMD and dimeric SLRPs. Blue background represents identical residues among SLRPs. Red squares indicate residues involved in the dimerization of DCN and the SLRP protein biglycan (BGN)

Recombinant OMD and DCN were highly purified as judged by SDS-PAGE (Fig. 1a). The molecular weight of OMD and DCN calculated from their amino acid sequences were 48.4 and 39.2 kDa, respectively, which corresponded reasonably well with the values determined from the position of the bands in SDS-PAGE (48 and 37 kDa, respectively, (Fig. 1a)). Because SLRPs, and DCN in particular, have been described generally as dimeric proteins^13^ we first investigated the oligomeric state of OMD in solution by size-exclusion chromatography with multi-angle light scattering (SEC-MALS). Contrary to our expectations, the data indicated that the molecular weight of OMD was 54.7 kDa, a value consistent with a monomeric conformation in solution. On the contrary, the size determined for DCN by SEC-MALS (60.6 kDa) was the result of a fast monomer–dimer equilibrium clearly favoring the dimeric form (Fig. 1b).

This finding could suggest that the structure of OMD is different from that of other SLRPs such as DCN. To address this question, we determined the X-ray crystal structure of OMD at 2.17 Å resolution (Fig. 1c; Table 1). The structure showed the typical curved solenoid fold characteristic of SLRPs displaying a large concave and convex surfaces. Despite the high concentration employed to crystalize the protein, OMD was found to be a monomer also in the crystalline state as demonstrated by the lack of stabilizing interactions and the small area of contact with neighboring protein chains in the crystal, as calculated with the PISA server. The structure of OMD was superimposed into the structure of DCN (Fig. 1d). The value of RMSD (calculated for the main chain alpha-carbons) was 1.3 Å, demonstrating that their overall fold was very similar. The difference in the dimerization propensity is explained by the lack of key intermolecular protein–protein interactions between neighboring units (Fig. 1e) caused by variations in their amino acid sequence (Fig. 1f). Collectively, our data have clearly evidenced that OMD exists as a stable monomer in solution.

**Table 1:** Data collection and refinement statistics

	Osteomodulin
Data collection	
Space group	P 1 2_1_ 1
Cell dimensions	
*a*, *b*, *c* (Å)	75.6, 110.7, 122.2
*α*, *β*, *γ* (°)	90, 107, 90
Resolution (Å)	35.2–2.17 (2.28–2.17)
*R*_merge_	0.084 (0.644)
*I*/*σ* (I)	9.2 (1.9)
Redundancy	3.6 (3.1)
Completeness (%)	99.2 (97.9)
Refinement statistics	
Resolution (Å)	35.2–2.17 (2.28–2.17)
No. of reflections	
*R*_work_/*R*_free_ (%)	21.7/24.5
No. of protein chains	4
No. of atoms	
Protein	10,014
Carbohydrate	182
Other	10
Water	315
B-factor (Å^2^)	
Protein	45.5
Carbohydrate	79.1
Others	63.5
Water	38.8
RMSD bond (Å)	0.015
RMSD angle (°)	1.73

The structure was determined from one crystal (the best diffracting crystal)

Values in parentheses are for the highest resolution shell

### Interaction between OMD and collagen molecules

The term “collagen” or “collagen molecules” refers to the soluble fraction of collagen after dialysis at neutral pH.

**Fig. 2:**
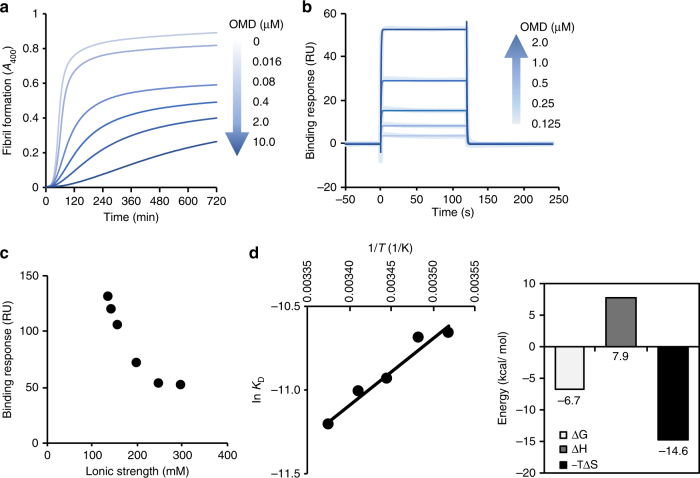
Interaction analysis between OMD and type-I collagen. **a** Fibril formation assay of type-I collagen with or without OMD in a dose-dependent manner. Gradient arrow indicates the concentration of OMD (darker was higher and lighter was lower, the same hereinafter). **b** Direct binding assay between OMD and type-I collagen using SPR. Type-I collagen was immobilized on a CM5 sensor chip. OMD was flowed on the chip in a dose-dependent manner (0.125–2 µM). **c** Salt-dependent binding level of OMD for type-I collagen in SPR measurement. **d** Thermodynamic parameters of the interaction between OMD and type-I collagen (right) based on van’t Hoff plot (left)

On the basis of our previous report^11^, we investigated the inhibitory effect of OMD in the formation of collagen fibrils. We observed that the rate of fibril formation decreased as the concentration of OMD was raised from 0.016 to 10 µM (Fig. 2a). A maximum inhibitory effect of ~100-fold was determined in the presence of 10 µM OMD when compared with the rates achieved in the absence of OMD (Table 2).

**Table 2:** Kinetic parameter of fibril formation in the presence of OMD or muteins

	*k*_obs_ (per h)						
	0 μM	0.016 μM	0.08 μM	0.4 μM	2 μM	10 μM	IC_50_ (μM)
OMD	0.96	0.81	0.48	0.31	0.18	0.01	0.08
E284R/E303R	0.86	0.93	0.88	0.81	0.67	0.79	0.29
chOMD	0.89	0.86	0.66	0.29	0.00	0.00004	0.22
chE284A	1.02	0.98	0.77	0.46	0.23	0.00003	0.32
chE284R	0.90	0.88	0.79	0.50	0.24	0.00028	0.61
chE303A	1.32	1.27	1.15	0.82	0.51	n.d.	0.37
chE303R	1.33	1.35	1.31	1.05	0.61	0.10	1.87
chE284R/E303R	1.00	1.00	0.94	0.87	0.82	0.65	2.18

Each 0 µM condition was the same component (1 µM collagen in PBS pH 7.4). The fibril formations at the concentration series were measured at the same time in each sample

Surface plasmon resonance (SPR) was employed to determine the binding affinity between OMD and collagen. OMD interacted with collagen with a fast association and dissociation steps (Fig. 2b). The dissociation constant (*K*_D_) was relatively weak, 25 µM. (Table 3). From these results, it was realized that the weak binding affinity of OMD to collagen is a necessary condition not to completely inhibit fibril formation, something that could otherwise occur if OMD engaged collagen with high affinity and low dissociation rates. We also show that OMD could bind to collagen fibrils during fibril formation as seen in images from immunoelectron microscopy (Supplementary Fig. 1). Overall, these data suggested that OMD could switch between bound–unbound state on collagen during fibril formation.

**Table 3:** Affinity parameter of the interaction between OMD, muteins, and collagen

	*K*_D_ (µM)	Binding (RU)
OMD	24.6	37.2
E284R/E303R	n.d.	−0.5
chOMD	25.4	117.8
chE173R	51.4	118.3
chE225R	29.1	105.6
chD254R	22.3	74.8
chD271R	25.6	71
chN278R	14.3	47.4
chE284R	32000	22.3
chE303R	72.4	3.7
chE311R	5.48	22.6
chE284R/E303R	n.d.	−5.5
chE284A	78.3	57.4
chE303A	n.d.	−0.5

*K*_D_ values were calculated by steady state analysis. Binding values were binding responses of 2 µM OMD or others injection onto collagen immobilized chip

The binding level of OMD to collagen gradually faded as the ionic strength increased from 137 to 300 mM (Fig. 2c), suggesting that electrostatic forces had a role in the interaction between OMD and collagen^14^. To rule out the possibility of salt-dependent conformational changes in OMD or collagen during the binding assays, the secondary structure and the thermal stability of OMD and collagen were examined by circular dichroism and differential scanning calorimetry (DSC). The absence of changes in the spectrum as a function of the ionic strength (Supplementary Fig. 2) and the unchanged values of *T*_M_ (Supplementary Fig. 3; Supplementary Table 1) supported the interpretation above, i.e., that electrostatic forces dominate the OMD–collagen interaction.

The thermodynamic parameters of binding were determined with the van’t Hoff equation using the temperature-dependent affinity constant determined by SPR^15^. At 32 °C the value of change of free energy (∆*G*) was –6.7 kcal/mol (corresponding to *K*_D_ = 12 µM), the change of enthalpy (∆*H*) was 7.9 kcal/mol, and the change of entropy (–*T*∆*S*) was –14.6 kcal/mol (Fig. 2d), indicating that the interaction between OMD and collagen was entropy-driven. Because collagen is typically hydrated in solution^16–18^, the favorable change of entropy can be explained by dehydration of the protein–protein interface upon binding of OMD^19^. We contemplated at least two scenarios that could explain entropy-driven dehydration, (i) the hydrophobic effect and (ii) the formation of salt-bridges. However, because the binding affinity was clearly reduced as a function of the ionic strength, we concluded that electrostatic interaction was the main driving force leading to the encounter and the binding of OMD to collagen. We corroborated this mechanism by an alternative SPR binding assay in which immobilized protein and analyte were exchanged (in this case OMD was immobilized, and collagen had the role of the analyte), reaching similar conclusions (Supplementary Fig. 4).

### Design of a chimeric OMD

**Fig. 3:**
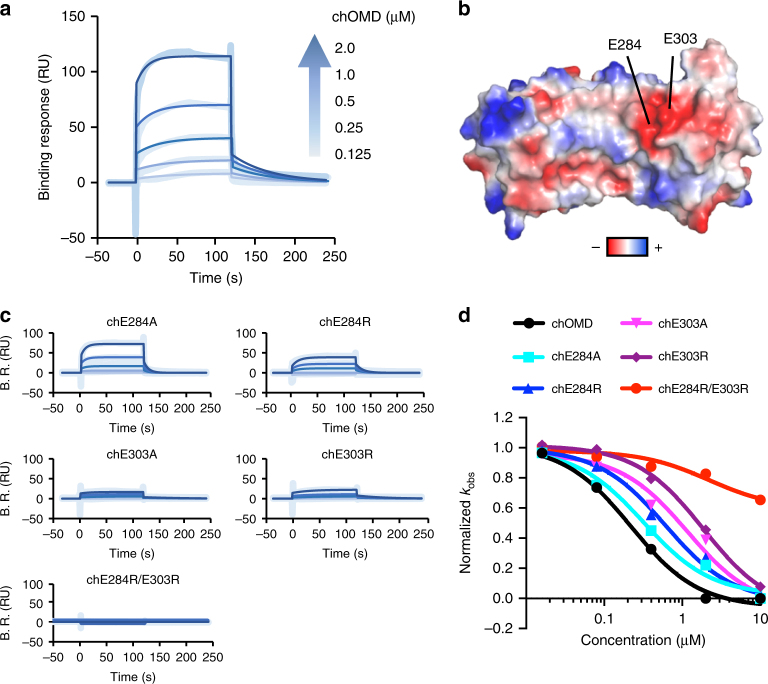
Determination of the collagen binding region of OMD using chimeric OMD. **a** Direct binding assay between chOMD and type-I collagen using SPR. Type-I collagen was immobilized on CM5 sensor chip. chOMD was flowed on the chip in a dose-dependent manner (0.125–2 µM). **b** Electrostatic potential of OMD region in chOMD (residues 117–364). The red and blue colors represent negative and positive electrostatic potentials, respectively. **c** Direct binding assay between mutants of chOMD and type-I collagen using SPR. Experiment was performed as in **a**. B.R. represent binding response. **d** Kinetic analysis of fibril formation with chOMD or mutants in a dose-dependent manner (0–10 µM)

We performed a site-directed mutagenesis study to determine the binding site of OMD to collagen. To improve the expression of recombinant OMD in *E. coli*, we employed a construct composed of a large portion of OMD and the N-terminal region of the leucine-rich repeat domain of internalin B (InlB), an approach previously reported for other proteins^20^. The chimeric OMD (chOMD) was designed by structural homology between OMD and InlB (Supplementary Fig. 5a). The construct chOMD was expressed in *E. coli* and purified by affinity chromatography followed by size-exclusion chromatography. From the SEC-MALS and functional data, it was concluded that chOMD was a monomer and fully functional protein similar to the parent OMD (Fig. 3a; Supplementary Fig. 5b–e), in contrast with the lack of binding activity for the purified InlB (Supplementary Fig. 5e).

Physicochemical properties of chOMD were determined by circular dichroism and DSC. The circular dichroism spectrum of chOMD showed negative peak around 218 nm characteristic of β-sheet (Supplementary Fig. 5f). However, the thermal stability of chOMD as judged from the value of TM was about 11 °C lower than that of OMD (Supplementary Fig. 5g). The weaker stability might reflect the influence of InlB (*T*_M_ = 30 °C) in the chimeric protein.

### Key residues of OMD for collagen binding

OMD is an acidic (negatively charged) protein with a pI of 5.2, whereas collagen is a basic (positively charged) protein with a pI of 9. Considering that the main driving force of binding was electrostatic in nature (see above), we mapped the electrostatic surface of OMD in the crystal structure searching for negatively charged patches that could contribute to binding (Fig. 3b). Various glutamic acid and aspartic acid residues of chOMD (Supplementary Fig. 6) were mutated to alanine. However, only the mutations E284A and E303A could be purified to satisfactory levels. For that reason, instead of Ala, we mutated the selected residues to arginine, and the affinity of the mutated protein for collagen was subsequently monitored by SPR.

Mutants chE284R and chE303R displayed lower binding affinities than chOMD (>1000-fold and 3-fold, respectively), whereas for chE311R a notable increase of affinity of c.a. 5-fold was observed (Fig. 3c, Table 3, and Supplementary Fig. 7a, b). However, we noted that the mutant chE311R was aggregation prone and even we detected some changes in its secondary structure (Supplementary Fig. 7c), leading to low binding levels that compromised the reliability of the measurement by SPR. No such deleterious effects were observed in chE284R and chE303R (Supplementary Fig. 7c; Supplementary Table 2). The role of these two residues was confirmed with Ala-substituted mutants (Fig. 3c). Predictably, the double mutant chE284R/E303R had completely lost the ability to bind to collagen (Fig. 3c; Supplementary Fig. 7a, b).

**Fig. 4:**
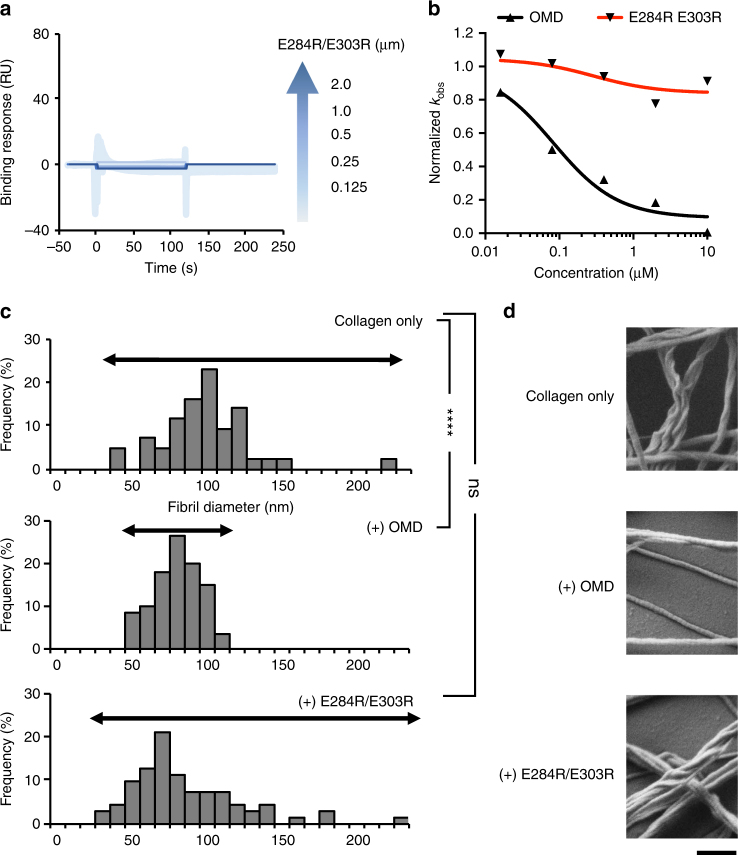
Loss of the regulatory effect of OMD for collagen fibrils by mutations of E284 and E303 of OMD. **a** Direct binding assay between E284R/E303R mutant of OMD and type-I collagen using SPR. **b** Kinetic analysis of fibril formation with E284R/E303R in a dose-dependent manner (0–10 µM). **c** Distribution of fibril diameter in the absence and presence of OMD or E284R/E303R. The data in the absence and presence of OMD were obtained from our previous study^11^. The values of diameter were obtained from TEM images. ****P-value < 0.001, ns not significant (*P*-value > 0.05). **d** Fibril structure in the absence and presence of OMD or E284R/E303R using SEM. Bar, 500 nm

These mutations had not only a negative impact on the affinity for collagen, but also on the capacity to inhibit fibril formation (Fig. 3d; Table 2). In agreement with the experiments above, a lack of inhibitory effect was maximal in the double mutant chE284R/E303R, in which the rates of fibril formation were little affected compared with collagen-alone, even at the highest concentration employed (10 μM). The circular dichroism spectra and thermal stability of the muteins were little affected, demonstrating a direct and specific effect of the substituted Glu residues (Supplementary Figs. 7, 8). Similar effects were observed with the double mutant E284R/E303R in the construct of full-length OMD, i.e., absence of binding to collagen as determined by SPR (Fig. 4a; Table 3) without loss of stability (Supplementary Fig. 8), and lacking the ability to reduce the rates of fibril formation (Fig. 4b; Table 2). We therefore concluded that residues Glu284 and Glu303 of OMD were essential for (i) collagen binding, and (ii) inhibiting collagen fibril formation.

### Regulation of fibril suprastructure by E284R/E303R

The impact of residues Glu284 and Glu303 on the shape and diameter of collagen fibrils was investigated. The diameter of collagen fibril was determined by transmission electron microscopy (TEM) and the morphological analysis by scanning electron microscopy (SEM). Whereas fibrils of collagen obtained in the presence of OMD had a defined (narrow) distribution, those obtained in the absence of OMD or in the presence of the double mutant E284R/E303R had a similar profile (*P*-value > 0.05; *F*-test) (Fig. 4c; Table 4). Similarly, the smoothing effect of OMD on the fibrils was lost in the double mutant, reverting to the twisted shape of collagen fibrils formed in the absence of the protein (Fig. 4d; Supplementary Fig. 9). However, the average diameter of fibrils in the presence of E284R/E303R (79.8 ± 36.1 nm; mean ± SD, standard deviation) was similar to that obtained in the presence of OMD (75.3 ± 15.7 nm, as demonstrated in our previous report^11^) (Table 4). These results clearly reveal a complex pattern in which electrostatic interactions driven by Glu284 and Glu303 govern critical, but not all, the effects of OMD on collagen fibril formation.

**Table 4:** Statistical evaluation of the distribution of fibril diameter in each condition

	Collagen only	(+) OMD
Average (nm)	94 ± 29.1	75.3 ± 15.6
Distribution	865.2	247
*P*-value	0.0000049 < 0.05	

	Collagen only	(+) E284R/E303R
Average (nm)	94 ± 29.1	79.8 ± 36.1
Distribution	865.2	1324.5
*P*-value	0.069 > 0.05	

Data of “Collagen only” and “(+) OMD” were obtained from our previous study^11^. Number of measured fibrils in the presence of E284R/E303R was 71 fibrils

## Discussion

Herein, we have revealed the structural features and mechanism of collagen activity of the protein OMD of the family of SLRPs. In particular, we have elucidated the molecular basis of the regulation of collagen fibril formation by OMD. At the core of this mechanism, we found that weak electrostatic forces between OMD and collagen mediate their interaction, leading to an optimal outcome in terms of the diameter and shape of the collagen fibrils produced (Supplementary Fig. 10). These weak electrostatic forces promote the right balance between sufficient binding levels and quick dissociation rates, thus allowing the growth of collagen fibrils with sufficient speed. Although there was a concern about the effect of FLAG-tag, we considered FLAG-tag of OMD did not affect the OMD–collagen interaction (Supplementary Fig. 11).

**Fig. 5:**
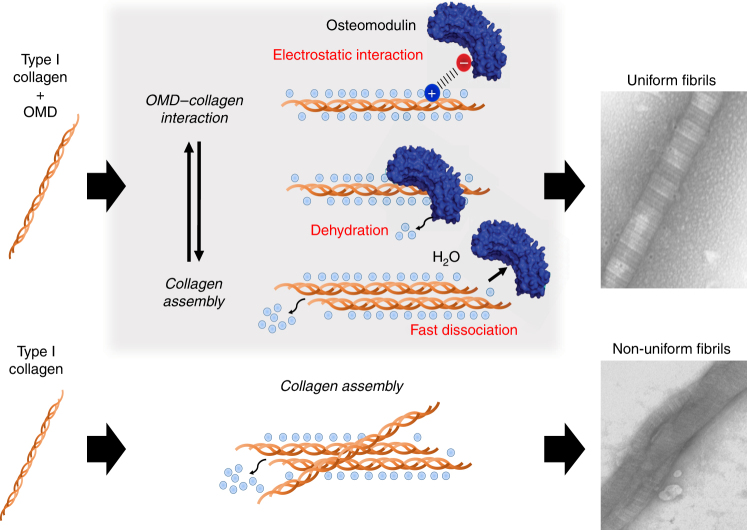
Model of the regulatory mechanism of OMD for generating uniform fibrils. OMD repeatedly binds and dissociates to type-I collagen during fibril formation in vitro. At first, OMD electrostatically binds to collagen. The driving force of the interaction is the favorable entropy caused by dehydration. After that, OMD dissociates from collagen, and then, collagen assembles itself. This phenomenon leads to a decrease the rate of fibril formation, and as a result, uniform fibrils are formed

In addition, the thermodynamic analysis suggested that the electrostatic OMD–collagen interaction was accompanied by dehydration at the interface^19^, which is consistent with the favorable change of entropy (Fig. 5). Importantly, these classes of interactions are critical for collagen–collagen interactions and for the conformation of fibrils, although the formation of collagen fibrils in vivo can be more complicated^21–24^. Residues Glu284 and Glu303 of OMD had an important role for the interaction between OMD and collagen. Electrostatic forces often act at long range between proteins^25^. Hence, the two residues seem to have a role for the attraction towards collagen. From our data, we cannot rule out that additional residues are involved in the interaction.

The novel mechanism proposed for OMD in this study has implications for the entire family of SLRPs^3,12^, shedding light on one of the key unknown aspects of tissue organization. We put forward the idea that in the absence of so-called governing proteins like OMD, collagen would assemble very fast in a non-orderly and aberrant fashion. However, when OMD is present, the rates of fibril formation slow down, reducing non-specific collagen–collagen interactions, and guiding the assembly of more uniform fibrils. This is at least partially achieved by reversibly binding to the surface of the growing collagen assembly^12^. We additionally propose that a change in the dissociation rate of OMD for collagen would influence the size of the fibrils. For example, we predict that OMD mutants with slow-dissociation rates will strongly inhibit the association of collagen resulting in thinner fibrils.

Because other SLRPs bind to type-I collagen, also decreasing the rate of fibril formation^11,26^, it is possible that the mechanism of OMD could also be applied to other SLRPs. For example, the SLRPs Fibromodulin displays negatively charged patches in its concave face like OMD, although in DCN they are less evident (Supplementary Fig. 12). Moreover, researchers have suggested that negative charged residues of SLRPs are involved in collagen binding^27–29^. These features are pointing to common mechanistic pathways, although the molecular details are expected to vary because of two reasons. One is the different nature of oligomerization for OMD (monomer) as opposed to that for DCN (equilibrium between monomer–dimer). Second, there are two binding sites for type-I collagen in DCN^27,30,31^, and the binding site of Fibromodulin is also unique^28,29,32^, which is different from OMD.

Some reports suggested SLRPs control the fibril size by binding to the surface of fibrils^3,12^. According to a docking simulation of DCN with collagen fibrils, the curvature of DCN could complement the fibril with a favorable energy^12^. The curvature of OMD might be acceptable to the surface of fibril, because the structural homology between OMD and DCN was almost identical (RMSD 1.3 Å, Fig. 1d). We note that residues E284 and E303 located on the concave side near the C-terminal region of OMD are critical for binding, whereas the region near the N-terminal end do not seem to critically contribute to collagen binding as suggested from mutations L144W and Q145A (Supplementary Fig. 13). The result suggests that the small part of OMD was critical for the binding to the collagen. Thus, we considered that specific binding to the surface of fibril was not a key factor for the regulation of fibrils by OMD.

In conclusion, we found that OMD binds to type-I collagen driven by weak electrostatic forces involving the residues Glu284 and Glu303 of a conspicuously, negatively charged patch of the protein. The weak nature of these interactions and the fast association and dissociation rates suggest that OMD shuttles on and off the growing collagen fibril in a fast-equilibrium. We propose that these weak interactions are critical factors governing the optimal growth of collagen fibrils in the presence of OMD, and would at least partially explain the function of other members of the SLRPs family.

## Methods

### Expression and purification

Expression and purification of human full-length OMD and muteins were performed as described previously^11^. Human OMD (without residues 1–20 corresponding to the signal peptide) with a C-terminal FLAG-tag was inserted into pFastbac1 vector. The signal peptide corresponded to the sp1–2 peptide^33^. A bacmid was made using vector transformed DH10Bac *E. coli* and baculovirus was constructed according to the commercial procedure of Bac-to-Bac® Baculovirus Expression System (Life technologies). Sf9 cells (purchased from Invitrogen) were infected with a baculovirus containing the sequence of OMD and incubated at 27 °C for 3 days, after which the cells were centrifuged and the supernatant collected. OMD was purified from the supernatant using an anti-FLAG M2 affinity gel (Sigma-Aldrich) followed by SEC in a Hiload 26/60 Superdex 200 pg column (GE Healthcare). The running buffer was PBS pH 7.4. Human full-length DCN was similarly produced. SDS-PAGE of DCN with marker shown in (Supplementary Fig. 14).

To produce the chimeric protein, *E. coli* BL21 (DE3) cells were transformed with a pET28b vector containing chOMD or muteins, and grown at 37 °C. Expression was induced with 1 mM isopropyl b-d-1-thiogalactopyranoside for 12 h, and cells collected by centrifugation at 8000×*g* for 10 min at 4 °C. The pellet was resuspended in buffer (20 mM Tris at pH 8, 500 mM NaCl, 20 mM imidazole), and lysed with an Ultrasonic Disrupter UD-201 instrument (TOMY). The lysate was centrifuged at 40,000×*g* for 30 min at 4 °C. The resulting supernatant was filtered with a DISMIC 28CP 0.8 µm unit (ADVANTEC, Tokyo, Japan), and applied onto a Ni-nitrilotriacetic acid agarose column (QIAGEN). The chOMD and muteins were eluted with a buffer composed of 20 mM Tris at pH 8, 500 mM NaCl, and 400 mM Imidazole. The protein was further purified by SEC in a Hiload 26/60 Superdex 200 pg column (GE Healthcare). The components of running buffer was 20 mM Tris at pH 8, 300 mM NaCl. When the proteins were measured with different buffer from the running buffer, they were dialyzed in the measurement buffer at 4 °C over night. Sequence of OMD, DCN, and chOMD were shown in (Supplementary Fig. 15).

Type-I collagen was purchased from Advanced BioMatrix. According to the description obtained from the manufacturer, the collagen (VitroCol) was naturally secreted from human neo-natal fibroblast cells and purified by enzyme treatment. The VitroCol collagen is stored at pH 2 that condition keep it triple helix monomers. The collagen was centrifuged at 15,000×*g* for 5 min at 4 °C and collected supernatant for each experiment after dialysis.

### SEC-MALS

SEC-MALS was performed at room temperature and using PBS buffer (pH 7.4). Proteins concentrated at 25 µM were loaded on a Hiload 10/300 Superdex 200 pg column (GE Healthcare). The elution of the proteins was monitored with a Dawn Heleos MALS detector (Wyatt Technology). Molecular weight of the proteins was calculated using ASTRA V software.

### Crystallization of OMD

Purified OMD (9.9 mg/ml) was screened in an Oryx8 automatic protein crystallization system (Douglas Instrument) with commercially available kits (Hampton Research) at 20 °C. Protein crystals were identified, and after optimization, the best crystals were obtained in a buffer containing 200 mM ammonium phosphate dibasic and 24% PEG 3350. Crystals were harvested after 3 weeks, immersed in mother liquor containing 40% PEG 3350, and frozen in liquid N_2_.

### X-ray data collection and structure determination

Data collection was carried out at beamline AR-NE3A at the Photon Factory (Tsukuba, Japan) at 1.000 Å and 100 K. Diffraction images were processed with the program MOSFLM^34^, and merged and scaled with the SCALA^35^. The structure was determined by the method of molecular replacement with the program PHASER^36^ using the coordinates of DCN (PDB entry code 1XKU)^13^. The models were refined with REFMAC^37^ of the CCP4 suite, and improved by manual inspection with COOT^38^. The quality of the refined structure was assessed with COOT and PROCHECK^39^. Of the 808 residues modeled one was found in the disallowed region of the Ramachandran plot (0.1%). Data collection and refinement statistics are given in Table 1.

### Fibril formation assay

Fibril formation assay was carried out according to a previous report^11^. Purified OMD, muteins, and type-I collagen were dialyzed against PBS (pH 7.4) for 16 h at 4 °C. OMD or muteins were mixed with collagen solution (final concentration of collagen was 1 or 0.8 µM (Supplementary Fig. 5d)) and then incubated for 12 h at 30 °C in a JASCO 700 spectrophotometer. Absorbance was continuously measured at 400 nm. Rate constants (*k*_obs_) of fibril formation was calculated by exponential fitting in a GraphPad Prism7. The rate constant at 0 µM condition (collagen only) of OMD or muteins was regarded as 1 (normalized *k*_obs_). Other rate constants were divided by the rate constant at the 0 µM condition. The normalized *k*_obs_ were plotted at the concentration series and the IC_50_ was calculated by exponential fitting in the software.

### Surface plasmon resonance

Interaction analysis between OMD (or muteins) and collagen was analyzed using SPR in a Biacore T200 instrument (GE Healthcare). A CM5 Biacore sensor chip (GE Healthcare) was used and activated by a treatment with *N*-hydroxysuccinimide/*N*-ethyl-*N*′-(3-dimethylaminopropyl) carbodiimide hydrochloride, followed by immobilization of collagen at around 8000 RU. The activated surface of the sensor was blocked with 1 M ethanolamine. The interaction between OMD (or muteins) and collagen was measured by injecting increasing concentration of OMD (or muteins) into the sensor chip at a flow rate of 30 µl/min. The measurement of the interaction was carried out in PBS (pH 7.4) containing 0.005% (v/v) Tween-20 at 20 °C. Collagen did not form fibrils at 20 °C (Supplementary Fig. 16).

Salt-dependent interaction analysis was performed in above buffer with the different concentration of sodium chloride. Binding response (Fig. 2c) was measured when 8 µM OMD was loaded into collagen immobilized chip. Another condition, which was OMD as ligand, was also measured (Supplementary Fig. 4). OMD was immobilized at around 3000 RU and collagen (0.125–3.5 µM) was injected into the sensor chip.

Data analysis was carried out with BIAevaluation software (GE Healthcare). Association (*k*_on_) and dissociation (*k*_off_) rate constants between OMD (or muteins) and collagen were calculated by a global fitting analysis assuming a Langmuir binding model and a stoichiometry of (1:1). The dissociation constant (*K*_D_) was calculated from the ration of the rate constants. However, there was too fast association between OMD and collagen, which could not allow the reliable calculation, except for the SPR data at OMD immobilized condition (Supplementary Fig. 4). Therefore, steady state analysis was performed to determine the dissociation constant (*K*_D_), which allows the calculation from the binding response at equilibrium (Req) and the analyte concentration.

### Analysis of thermodynamic parameters

Thermodynamic parameters; change in enthalpy (∆*H*°) and entropy (∆*S*°) were calculated from the slope and intercept of the temperature dependence of the dissociation constant using the van’t Hoff formula:

$$\ln \;K_{\mathrm{D}} = -\Delta H^\circ /RT + \Delta S^\circ /R,$$

where *R* is the gas constant and *T* is the absolute temperature.

### Transmission electron microscopy

The data corresponding to Fig. 4c and Table 4 obtained with only collagen or with collagen and WT OMD correspond to a previous study of our group^11^. Preparation of E284R/E303R was also performed in the same way of the paper^11^. In brief, E284R/E303R (final concentration was 0–10 µM) were mixed with collagen (final concentration was 0.8 µM) in PBS (pH 7.4) and incubated for 12 h at 37 °C. The samples were adsorbed on the membrane formed on the surface of cupper grids for 5 min at 37 °C and then excess fluid was blotted with filter paper and stained with 1% uranyl acetate. Images of samples were obtained by Hitachi H-7500 electron microscope. Diameter analysis was carried out using Image J software.

### Scanning electron microscopy

Samples for SEM analysis at the three conditions (Collagen only, presence of OMD and E284R/E303R) were prepared and measured at the same time according to ref.^11^. At first, fibril formation was carried out with the same method as TEM analysis. The fibril formed samples were adsorbed on super smooth silicon wafer (EM Japan) for 5 min followed by fixation with 1% glutaraldehyde in 0.1 M phosphate buffer for an hour at room temperature. The specimens were washed by phosphate buffer three times and post fixed with 1% OsO_4_ in phosphate buffer for an hour on ice. They were dehydrated with a graded series of ethanol followed by t-butanol replacement. Then, samples were freeze dried using ES-2030 freeze dryer (Hitachi High-Technologies) and coated with OsO_4_ using HPC-1S osmium coater (Vacuum Device). SEM was performed with Zeiss SIGMA scanning electron microscope.

### Immunoelectron microscopy

Collagen was incubated for 0 min, 10 min and 12 h at 37 °C in PBS pH 7.4. These samples were adsorbed on Ni grid for 5 min. OMD was put on the grids and left for 30 min at room temperature After washing the grids three times by PBS, anti-OMD mouse antibody was put on the grids and incubated for 30 min at room temperature. The antibody was produced in our laboratory and verified the binding ability to OMD by ELISA, Western blot, and SPR. After washing the grids three times by PBS, anti-mouse IgG antibody conjugated with 5-nm gold particles was put on the grids and incubated for 20 min at room temperature. After immunostaining, the samples were stained with 1% uranyl acetate and examined by TEM.

### Design of chimeric OMD

Chimera OMD was constructed in reference to the method of the design of the Repebody scaffold^40^. First, alignment analysis of LRR pairs between OMD and InlB was carried out using the program GENETYX. Several candidates were chosen from homology of the LRR pairs. Second, the candidates of OMD were superimposed to the candidates of InlB using the program Pymol. We determined the connecting residue of InlB and OMD from the superimposed structures. A fusion gene was constructed with the in-fusion method. Human OMD without signal sequence (corresponding to residues 21–421) cloned in pET28b vector was used as a template. The inserted sequence of InlB and the deleted sequence of OMD was amplified using PCR. They were fused and transformed into JM109 cells. The sequence of the designed chOMD was confirmed by DNA sequencing.

### Circular dichroism

Circular dichroism spectra of samples were measured at 20 °C using JASCO J-820 spectropolarimeter in a 1 mm path-length quartz cell. Samples were prepared at 10 µM in PBS (pH 7.4).

### DSC

DSC measurements were performed using an automated VP-DSC microcalorimeter (VP-Capillary DSC, GE Healthcare). Samples were prepared at 10 µM and heated from 10 to 100 °C at a rate of 60 °C/h. Analysis of DSC was carried out using a two-state model with ORIGIN7 software. Each sample were expressed, purified and measured at the same time in every figure or table.

### Data availability

Coordinates and structure factors for the structure of osteomodulin were deposited in the Protein Data Bank under accession code: 5YQ5. We confirm all relevant data are available from us and Figshare (DOI: 10.6084/m9.figshare.5956066).

## Electronic supplementary material

Supplementary Information
