# Supplementary material for: Molecular basis for governing the morphology of type-I collagen fibrils by Osteomodulin
Source: Commun Biol. 2018 Apr 19;1:33. doi: 10.1038/s42003-018-0038-2 (PMC6123635; doi:10.1038/s42003-018-0038-2)
Supplement: Supplementary file 1 — Supplementary Information [file 42003_2018_38_MOESM1_ESM.pdf]

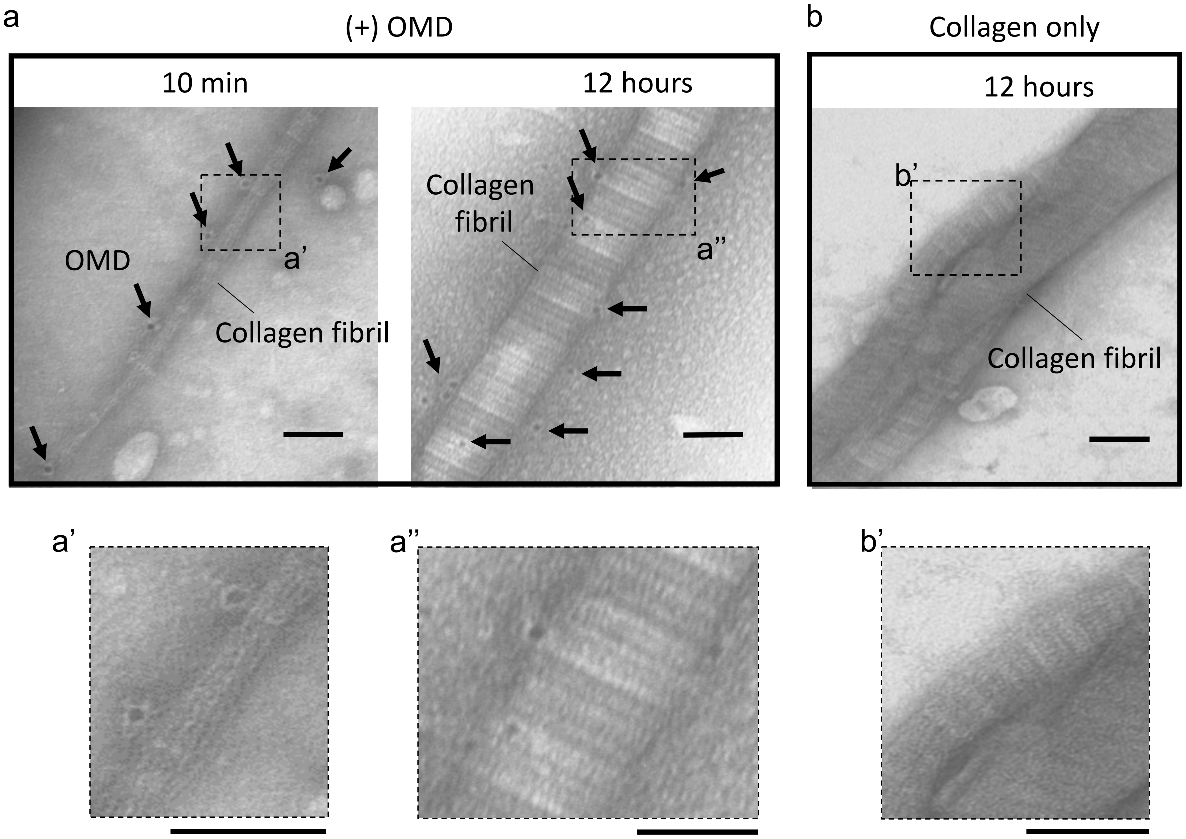

Supplementary Figure 1. Localization of OMD on collagen fibrils using immunoelectron microscopy.

(a) Collagen fibril in the presence of OMD after 10 min incubation and after 12 hours incubation at 37 °C. Black arrows represent localization of OMD. OMD was detected by anti-OMD mouse antibody and anti-mouse IgG antibody conjugated with gold particles. **(a’)** and **(a”)** correspond to the enlarged images of the dotted square regions of 10 min and 12 hours incubated condition of

(a). **(b)** Collagen fibril in the absence of OMD after 12 hours incubation at 37 °C. Bar corresponds to 50 nm. **(b')** corresponds to the enlarged image of the dotted square region of **(b)**.

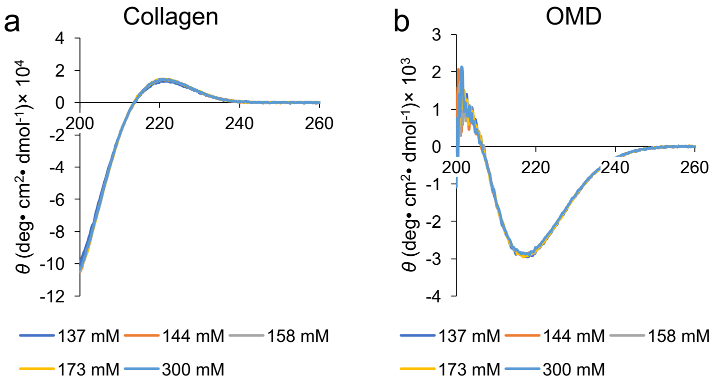

Supplementary Figure 2. CD spectra of collagen and OMD as a function of the ionic strength.

CD spectra of **(a)** collagen and **(b)** OMD at 20 °C in each condition of ionic strength. The spectra were recorded between 200 and 260 nm.

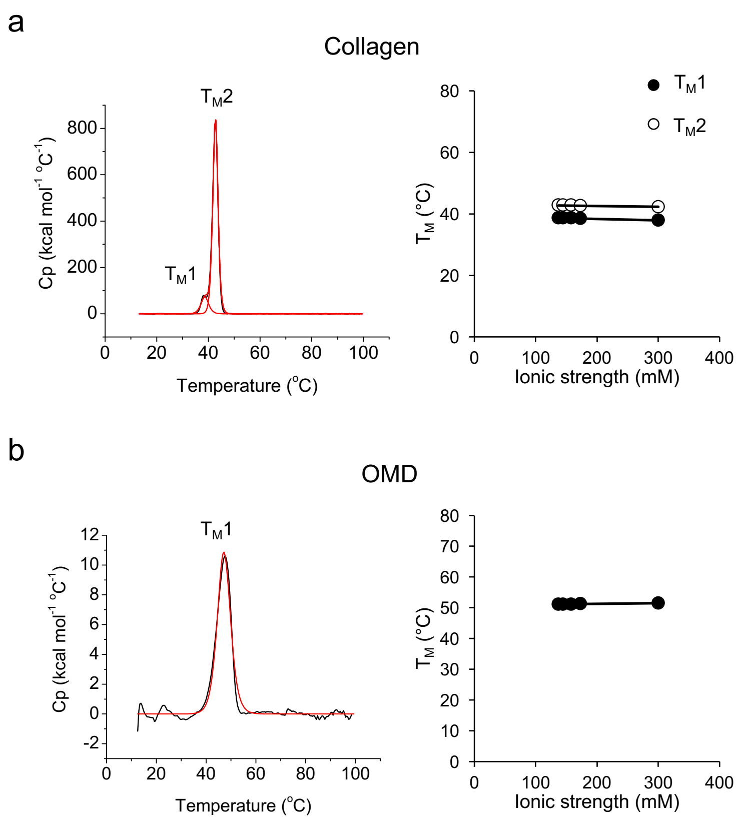

Supplementary Figure 3. Thermal stability of collagen and OMD as a function of the ionic strength using DSC.

DSC data of **(a)** collagen and **(b)** OMD. **(a)** There were two peaks, T_M1 and T_M2. The values scarcely changed in the range of 137-300 mM of ionic strength for collagen (a) or OMD (b).

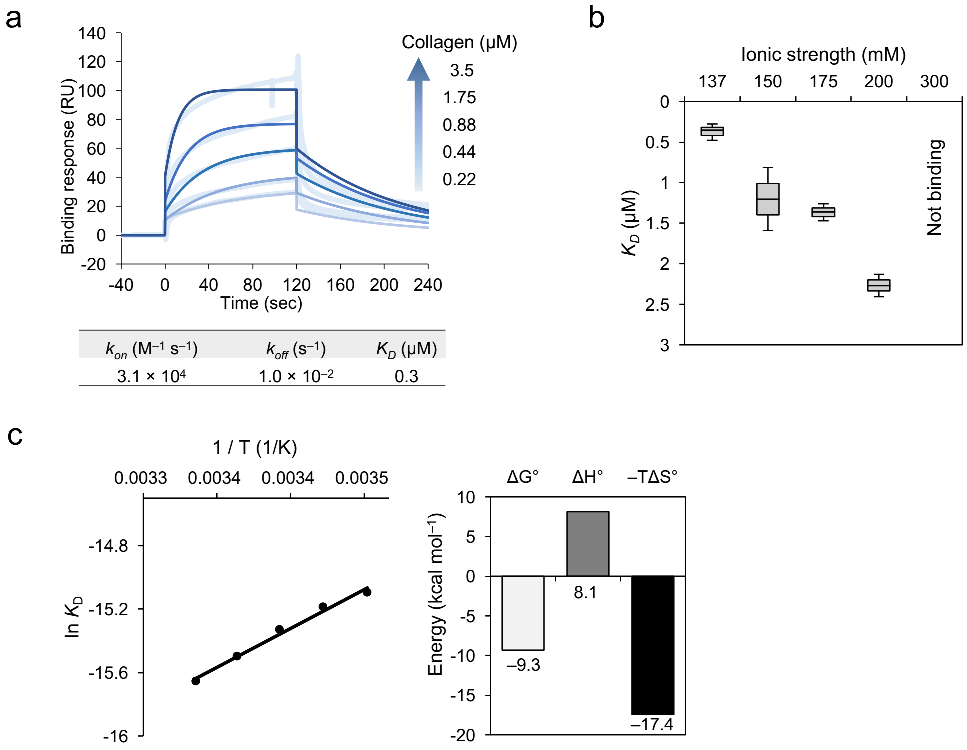

Supplementary Figure 4. Interaction analysis between OMD and collagen.

(a) Interaction analysis between OMD and collagen using SPR. OMD was immobilized on CM5 sensor chip and collagen was injected as an analyte in a dose dependent manner. Kinetic parameters were calculated with BIAevaluation software. **(b)** Box-and-whisker plot of salt-dependent affinity between OMD and collagen. The affinity was calculated from the same way as in panel **(a)**. The kinetic parameters could not be obtained at 300 mM of ionic strength because of the insufficient binding response. The experiments in each condition were performed 3 times

(137 mM) and 2 times (other conditions). **(c)** Thermodynamic parameters of the interaction between OMD and collagen calculated from SPR experiments in which OMD was immobilized and collagen molecules were injected at 109 nM~875 nM.

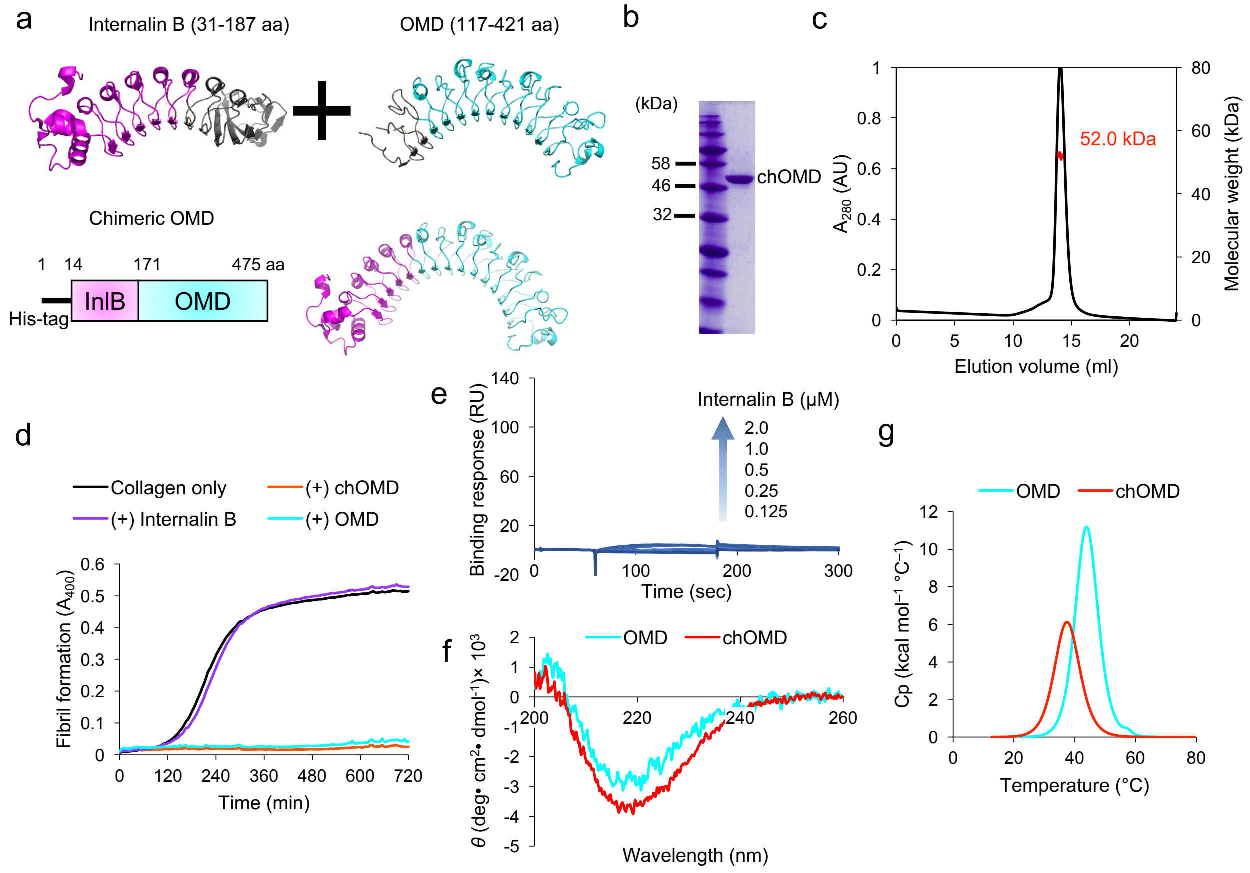

Supplementary Figure 5. Design and analysis of collagen binding of chOMD.

(a) Structural model of the chimeric OMD. Structural data of Internalin B was obtained from PDB. **(b)** SDS-PAGE of chOMD after purification. **(c)** SEC-MALS of 100 μM chOMD. Black line represents absorbance 280 nm and red dotted line represents molecular weight. **(d)** Fibril formation analysis in the absence or presence of chOMD, internalin B or OMD at 10 μM at 30 ˚C. **(e)** Interaction analysis between internalin B and collagen using SPR in which collagen was immobilized. **(f)** CD spectra of OMD and chOMD. Both spectra showed negative peak around

218 nm which suggested beta-sheet specific structure. **(g)** Thermal stability of OMD and chOMD using DSC.

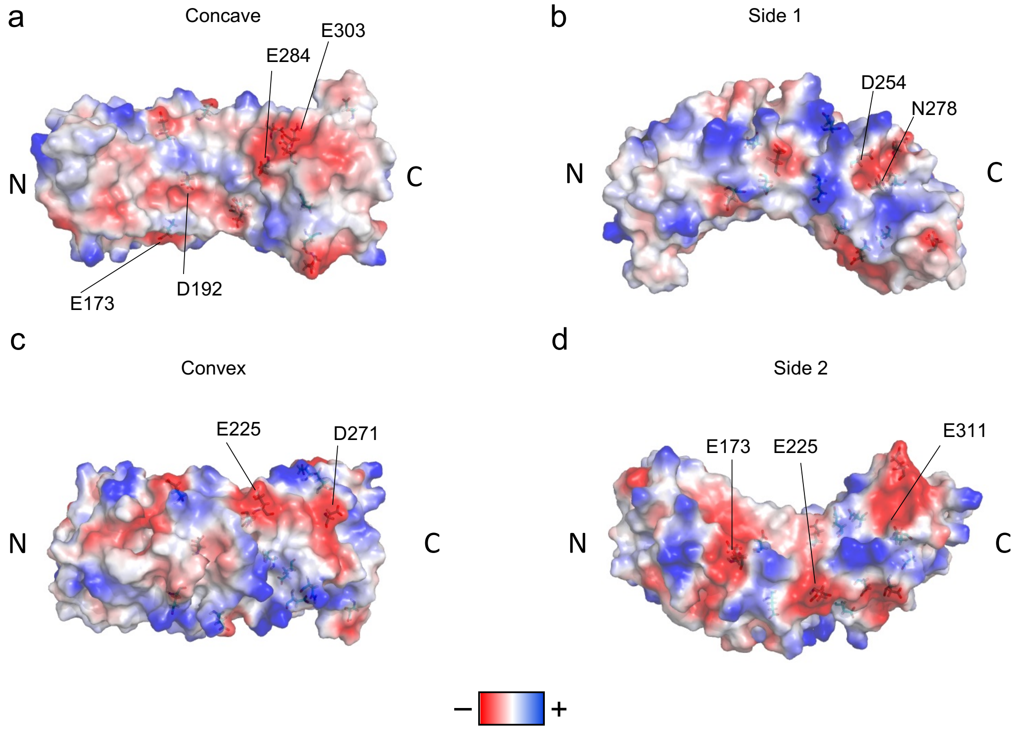

Supplementary Figure 6. Mutation study of chOMD for the determination of collagen binding site.

(a-d) Electrostatic potential of OMD using Pymol software. **(a)** Concave face, **(b)** Side 1, **(c)** Convex face and **(d)** Side 2 of OMD.

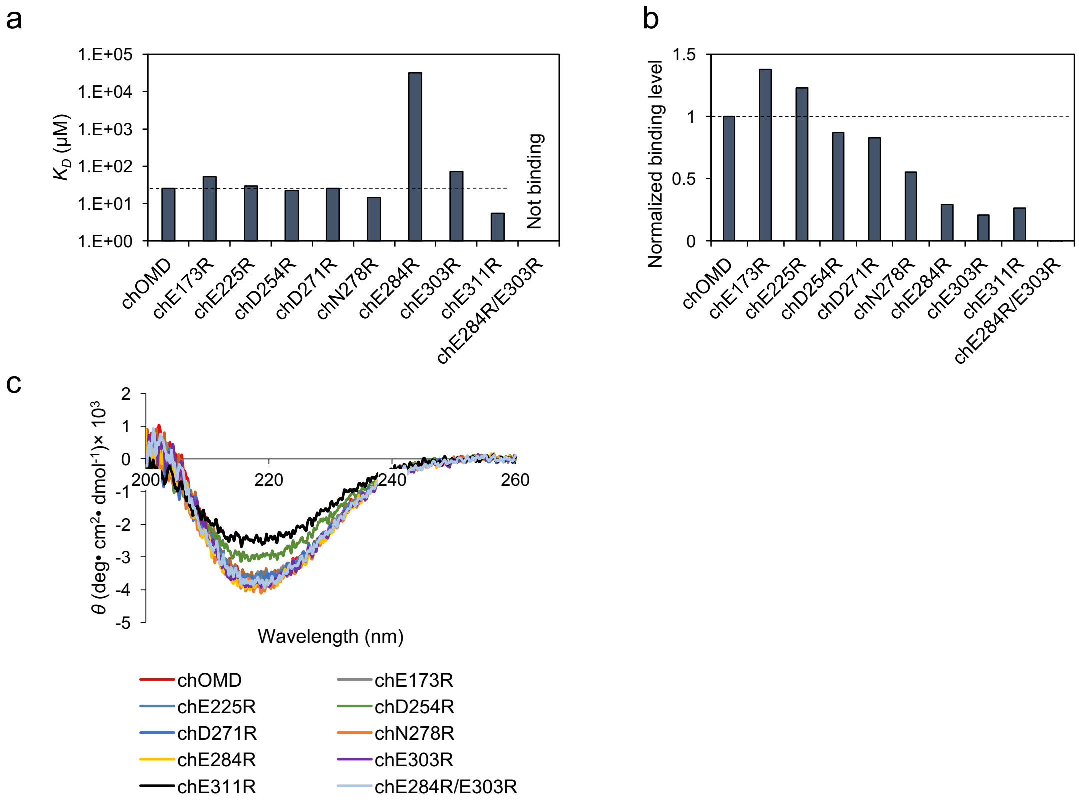

Supplementary Figure 7. Interaction analysis between chimeric mutants and collagen.

(a) Affinity of chOMD (or chOMD mutants) for collagen monitored by SPR and calculated with the affinity analysis module in the BIAevaluation software. **(b)** Normalized binding level for collagen using SPR. Binding level of chOMD was regarded as 1.0. **(c)** CD spectra of chOMD and the mutants.

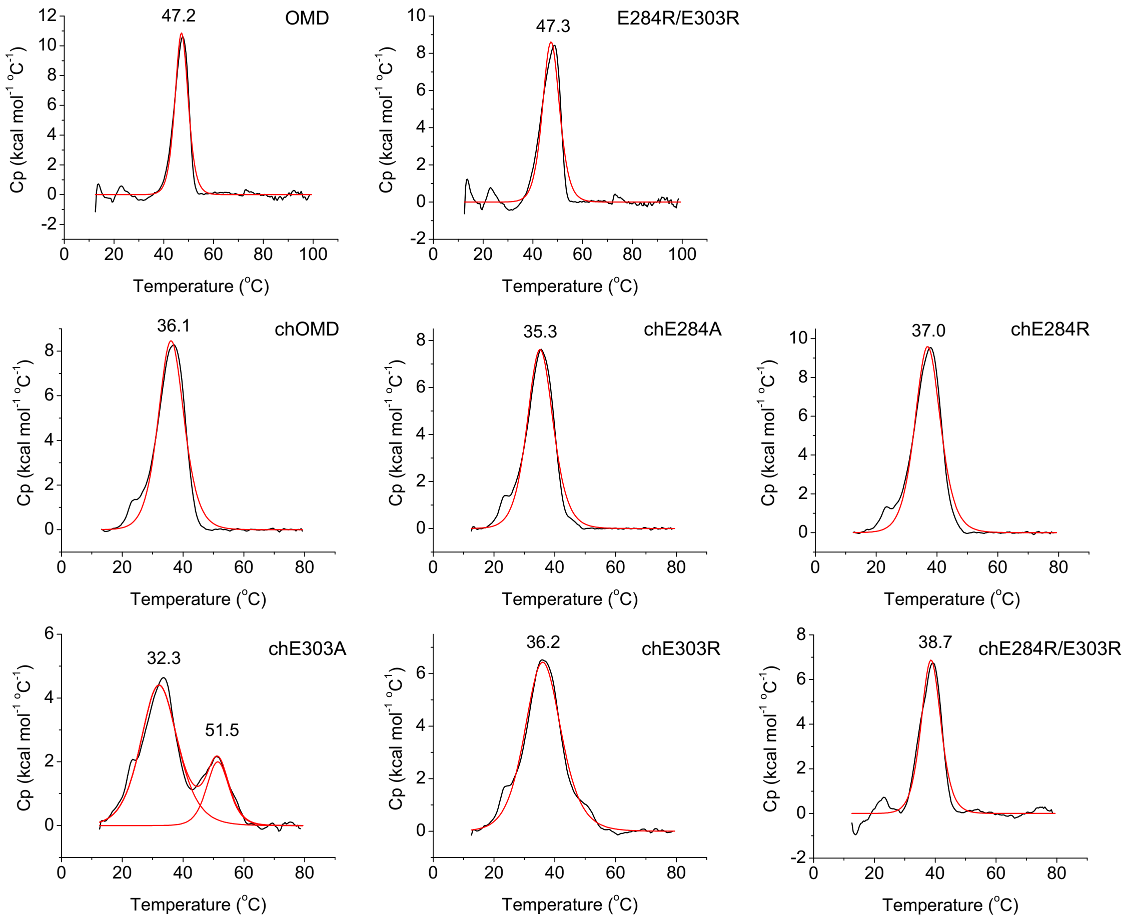

Supplementary Figure 8. Thermal stability of OMD and OMD mutants using DSC.

a

Collagen only

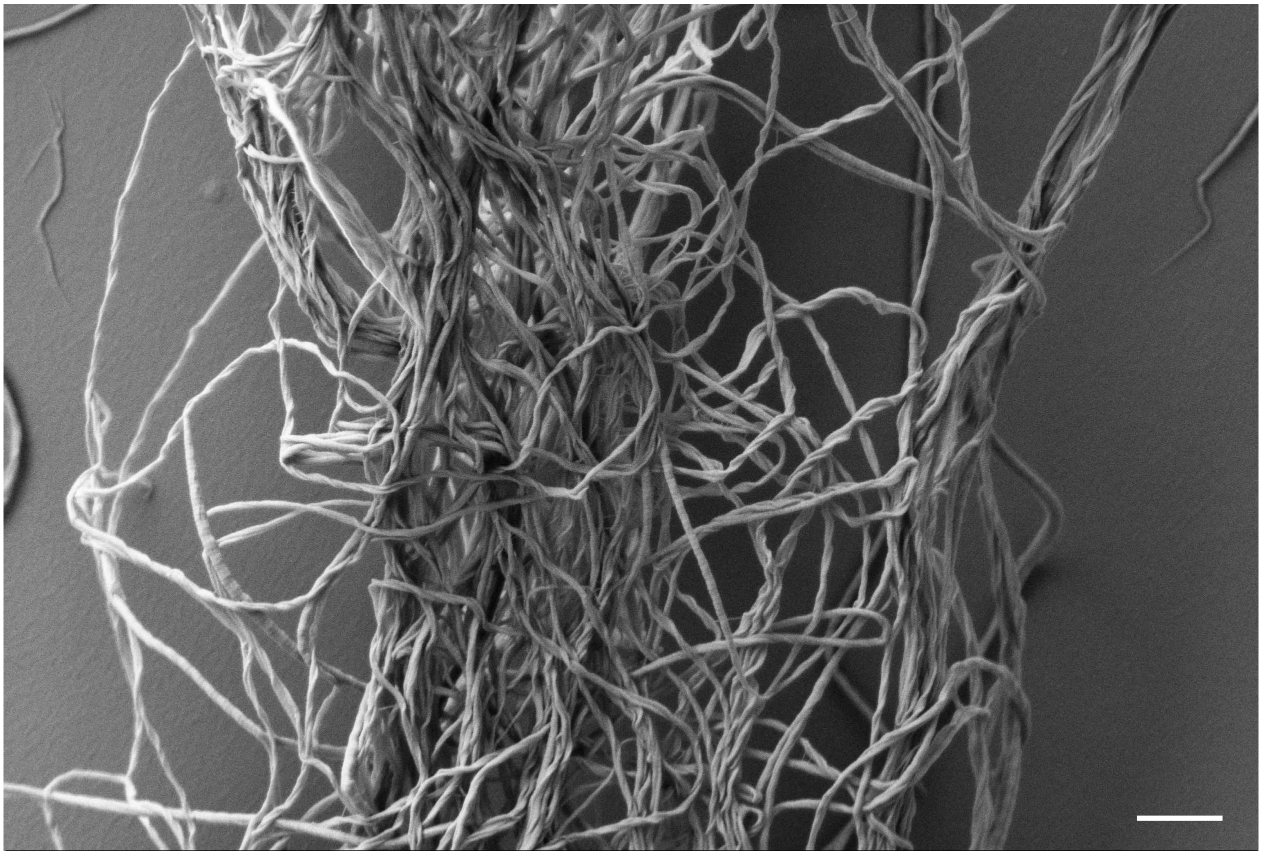

56 b

(+) OMD

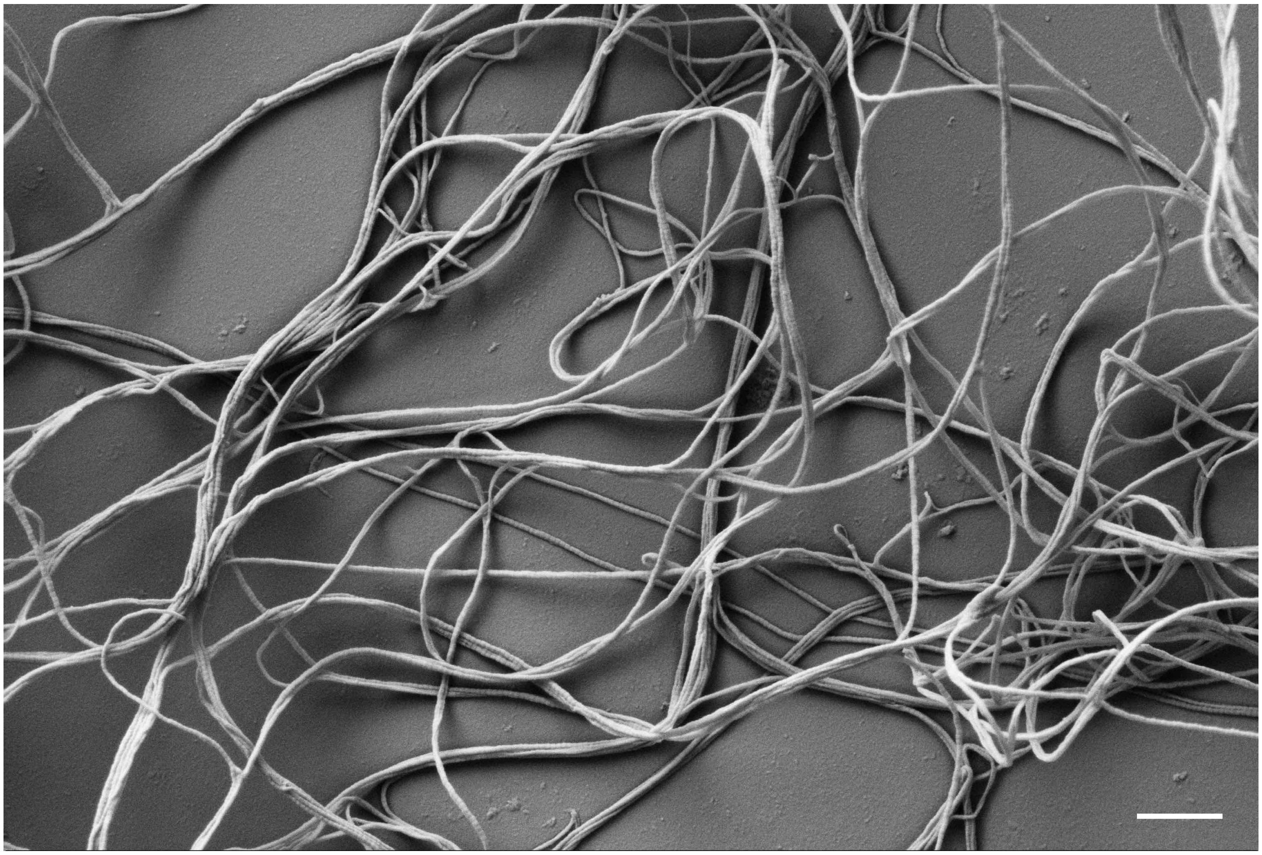

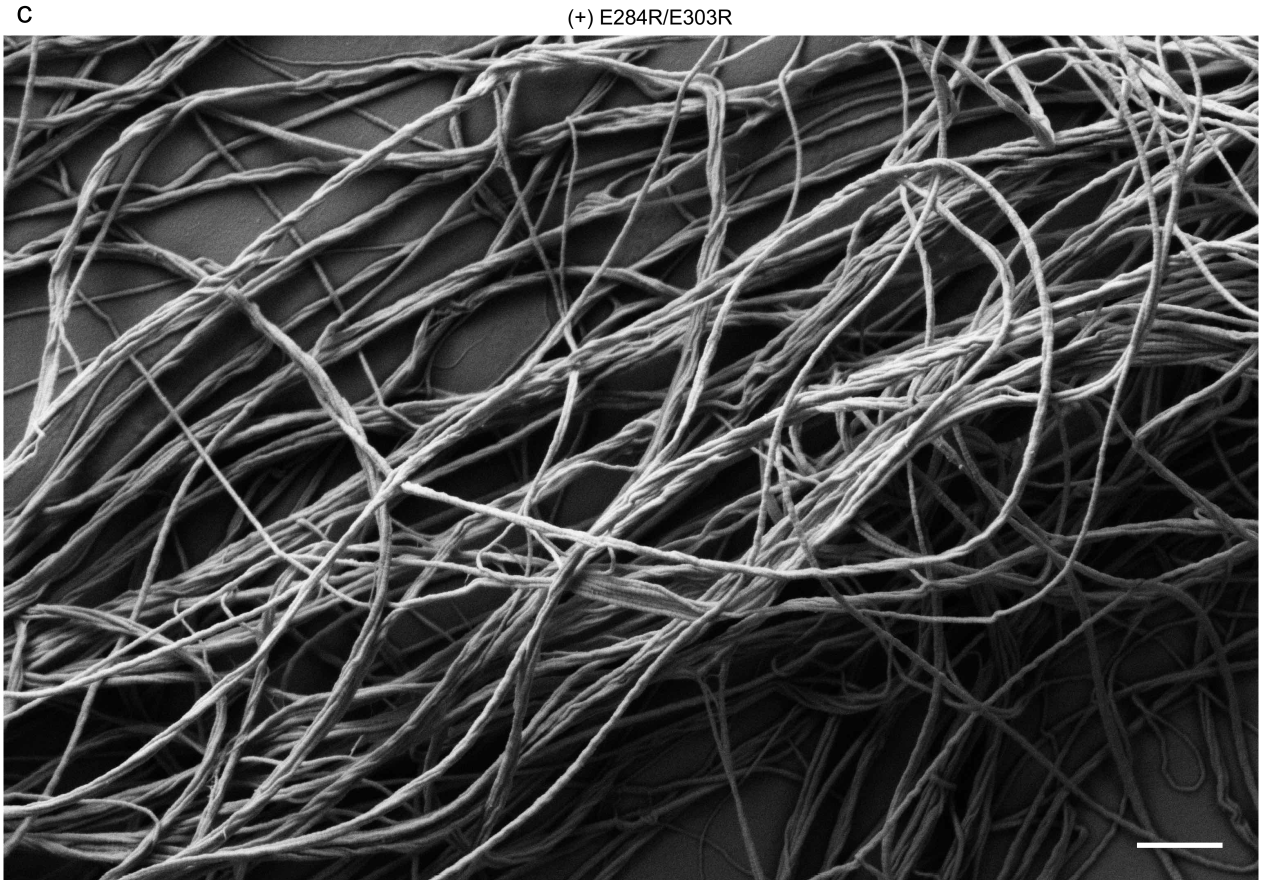

Supplementary Figure 9. SEM images of collagen fibrils.

(a) Collagen only, **(b)** collagen with OMD and **(c)** collagen with E284R/E303R. Magnification of these images was 20k. Bars, 1 μm (**a**-**c**).

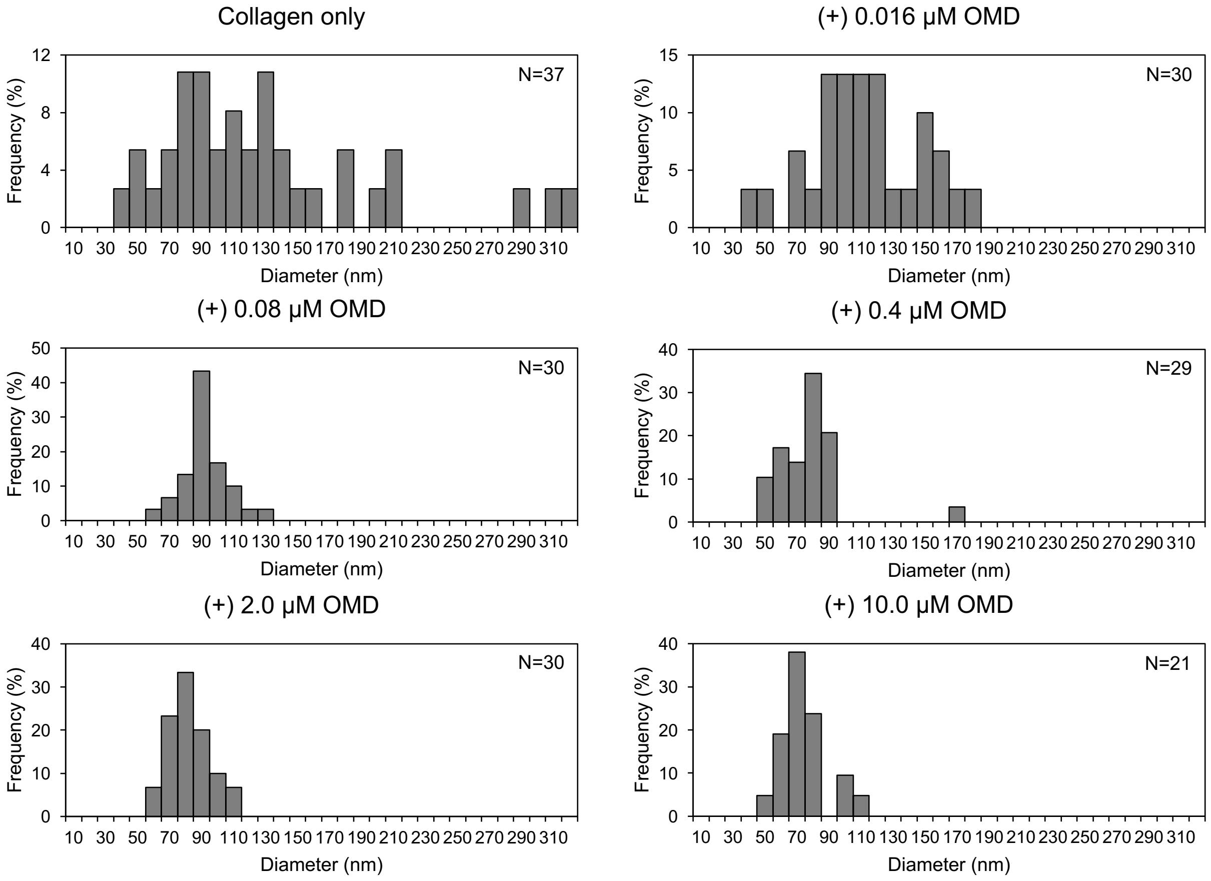

Supplementary Figure 10. Distribution of fibril diameter in the presence of increasing concentrations of OMD.

Fibril formation analysis was performed at the following condition. Final concentration of collagen and OMD was 0.8 μM and 0~10 μM respectively. They were incubated at 37 ˚C for 12 hours and then, were observed using TEM. “N” represents number of measured fibrils. Fibril diameter was measured using Image J.

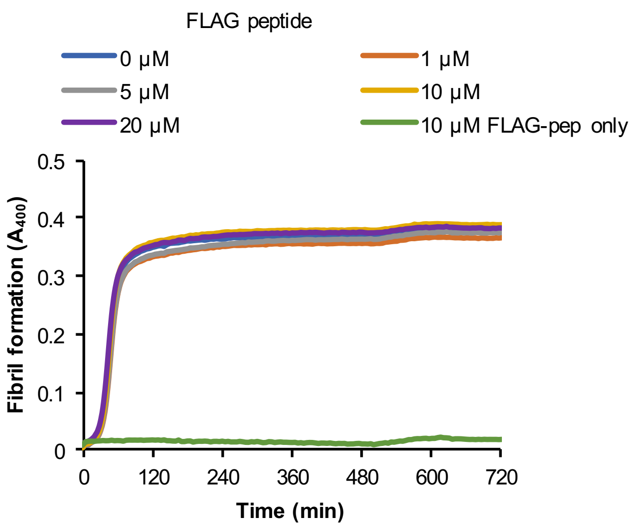

Supplementary Figure 11. Collagen fibril formation in the presence of FLAG-peptide.

Type I collagen was dialyzed against PBS (pH 7.4) for 16 hours at 4 °C. FLAG-peptide was dissolved with PBS and mixed with a collagen solution (Final concentration of collagen was 0.8 µM) and then incubated for 12 hours at 37 °C in a JASCO 700 spectrophotometer. Absorbance was monitored at 400 nm

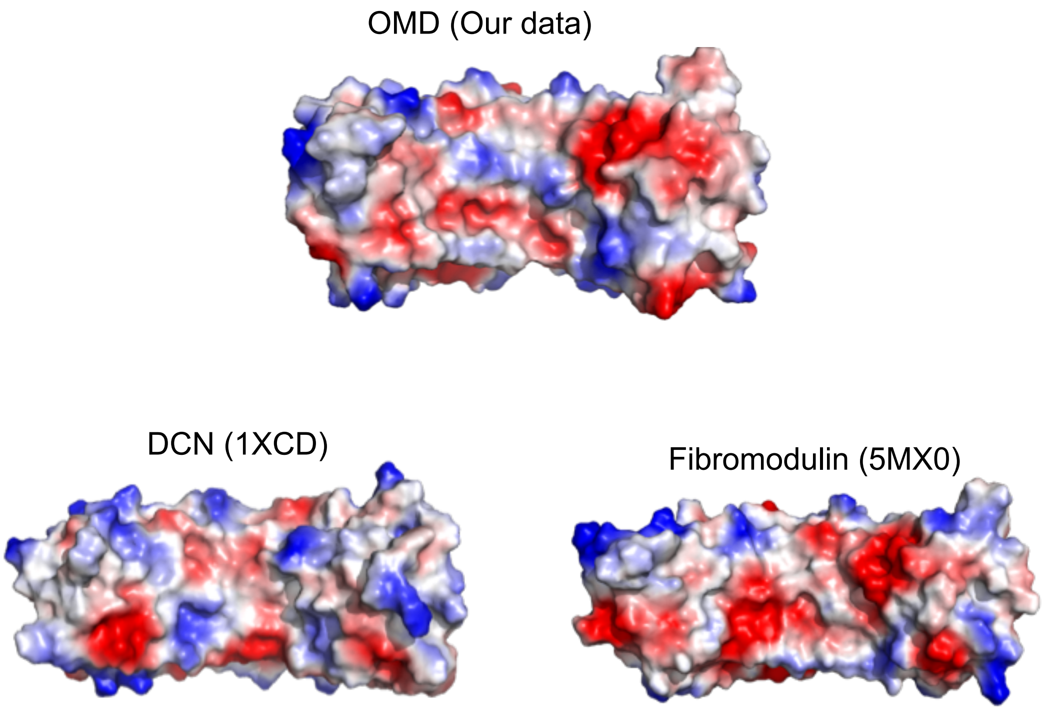

Supplementary Figure 12. Electrostatic potentials of OMD, DCN and Fibromodulin.

Red and blue color represents negative and positive charge, respectively.

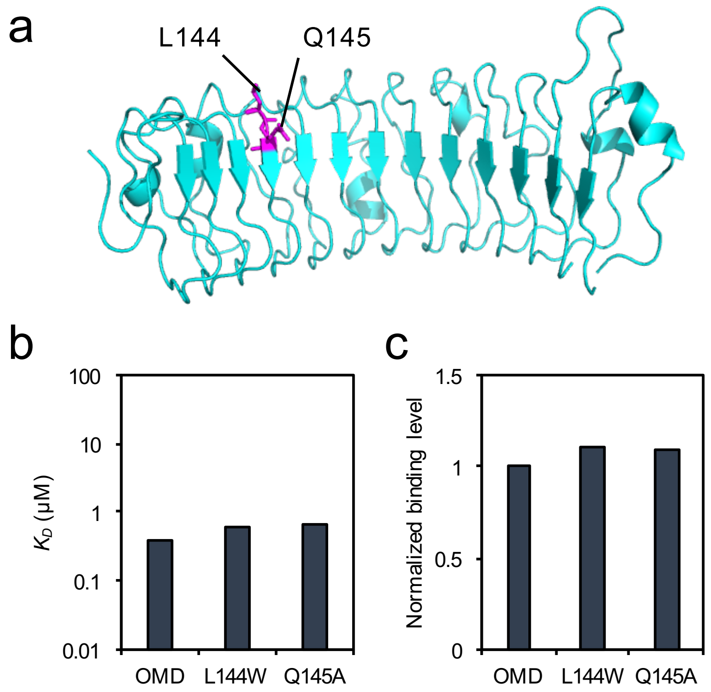

Supplementary Figure 13. Binding of OMD mutants to collagen molecules.

(a) The location of residues L144 and Q145 in the structure of OMD. **(b)** The affinity of OMD, L144W, and Q145A for collagen molecules. OMD, L144W and Q145A were respectively immobilized at 5453 RU, 5247RU and 5884 RU. And then, collagen molecules were injected at the concentrations series (0.2 μM to 3.5 μM). The affinity was calculated from steady state analysis. **(c)** Normalized binding level of OMD, L144W and Q145A for collagen molecules. Binding level was measured at 3.5 μM collagen molecules injected. The value was divided by the immobilization level because the immobilization level of OMD and muteins was different. And the binding level of OMD was regarded as 1, representing normalized binding level.

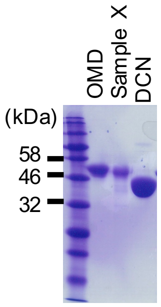

Supplementary Figure 14. Full-size SDS-PAGE gel shown in Figure 1a.

We show the complete SDS-PAGE gel appearing in Figure 1a. From left, molecular weight marker, purified OMD, Sample X (not related to this manuscript), and purified DCN.

>OMD-FLAG

MRVLVLLACLAAASNAGSQYETYQWDEDYDQEPDDDYQTGFPFRQNVDYGVPFHQYTLGCV
SECFCPTNFPSSMYCDNRKLKTIPNIPMHIQQLYLQFNEIEAVTANSFINATHLKEINLSHNKIKSQ
KIDYGVFAKLPNLLQLHLEHNNLEEFPFPLPKSLERLLLGYNEISKLQTNAMDGLVNLTMLDLCY
NYLHDSLLKDKIFAKMEKLMQLNLCSNRLESMPPGLPSSLMYLSLENNSISSIPEKYFDKLPKLH
TLRMSHNKLQDIPYNIFNLPNIVELSVGHNKLKQAFYIPRNLEHLYLQNNEIEKMNLTVMCPSIDP
LHYHHLTYIRVDQNKLKEPISSYIFFCFPHIHTIYYGEQRSTNGQTIQLKTQVFRRFPDDDDESED
HDDPDNAHESPEQEGAEGHFDLHYYENQEAAADYKDDDDK

>Decorin-FLAG

MRVLVLLACLAAASNAGSGPFQQRGLFDFMLEDEASGIGPEVPDDRDFEPSLGPVCPFRCQC
HLRVVQCSDLGLDKVPKDLPPDTTLLDLQNNKITEIKDGDFKNLKNLHALILVNNKISKVSPGAFT
PLVKLERLYLSKNQLKELPEKMPKTLQELRAHENEITKVRKVTFNGLNQMIVIELGTNPLKSSGIE
NGAFQGMKKLSYIRIADTNITSIPQGLPPSLTELHLDGNKISRVDAASLKGLNNLAKLGLSFNSIS
AVDNGSLANTPHLRELHLDNNKLTRVPGGLAEHKYIQVVYLHNNNISVVGSSDFCPPGHNTKKA
SYSGVSLFSNPVQYWEIQPSTFRCVYVRSAIQLGNYKAAADYKDDDDK

>chOMD

MGSSHHHHHHGSGAKVQAETITVPTPIKQIFSDDAFAETIKDNLKKKSVTDAVTQNELNSIDQIIA
NNSDIKSVQGIQYLPNVTKLFLNGNKLTDIKPLANLKNLGWLFLDENKVTDLSSLKDLKKLKSLSL
EHNGISDINGLVHLPQLESLYLGNNKITDITVLSRLTKLKEINLSHNKIKSQKIDYGVFAKLPNLLQL
HLEHNNLEEFPFPLPKSLERLLLGYNEISKLQTNAMDGLVNLTMLDLCYNYLHDSLLKDKIFAKM
EKLMQLNLCSNRLESMPPGLPSSLMYLSLENNSISSIPEKYFDKLPKLHTLRMSHNKLQDIPYNI
FNLPNIVELSVGHNKLKQAFYIPRNLEHLYLQNNEIEKMNLTVMCPSIDPLHYHHLTYIRVDQNKL
KEPISSYIFFCFPHIHTIYYGEQRSTNGQTIQLKTQVFRRFPDDDDESEDHDDPDNAHESPEQE
GAEGHFDLHYYENQE

Supplementary Figure 15. Amino acid sequence of OMD, DCN and chOMD

Red: signal peptide (sp1-2). Gray: Linker. Blue: FLAG-tag. Orange: His-tag. Pink: segment of internalin B.

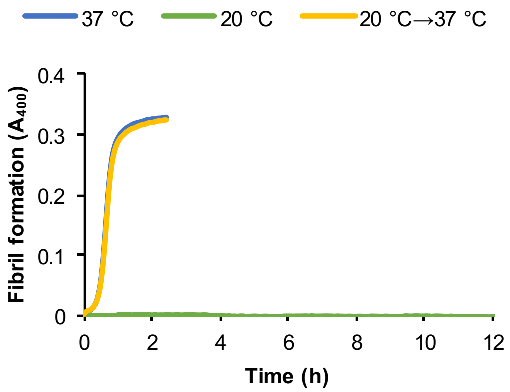

Supplementary Figure 16. Collagen fibril formation at different temperatures.

The concentration of molecules of type I collagen was 0.8 μM. Samples were incubated at 20 °C or at 37 °C. When the temperature was increased from 20 °C to 37 °C, the collagen molecules were first incubated at 20 °C for 12 hours.

Supplementary Table 1. Thermal stability of collagen and OMD as a function of the ionic strength using DSC.

Ionic strength	137 mM		144 mM		158 mM		173 mM		300 mM	
	T_M 1	T_M 2	T_M 1	T_M 2	T_M 1	T_M 2	T_M 1	T_M 2	T_M 1	T_M 2
Collagen	38.6	42.7	38.6	42.7	38.6	42.7	38.4	42.6	37.9	42.3
OMD	51.1	-	51.2	-	51.2	-	51.3	-	51.5	-

Supplementary Table 2. Thermal stability of chimeric OMD and the mutants using DSC.

	T_M (°C)
chOMD	42.5
chE173R	42.3
chE225R	41.8
chD254R	37
chD271R	38.2
chN278R	38.5
chE284R	42.3
chE303R	43.3
chE311R	n.d.
chE284R/E303R	43.8
